# The relationship between eye movement and vision develops before birth

**DOI:** 10.3389/fnhum.2014.00775

**Published:** 2014-10-02

**Authors:** Veronika Schöpf, Thomas Schlegl, Andras Jakab, Gregor Kasprian, Ramona Woitek, Daniela Prayer, Georg Langs

**Affiliations:** ^1^Division of Neuro- and Musculoskeletal Radiology, Department of Biomedical Imaging and Image-guided Therapy, Medical University of ViennaVienna, Austria; ^2^Computational Imaging Research Laboratory, Department of Biomedical Imaging and Image-guided Therapy, Medical University of ViennaVienna, Austria; ^3^Computer Science and Artificial Intelligence Lab, Massachusetts Institute of TechnologyCambridge, MA, USA

**Keywords:** functional connectivity, development, eye movement, ICA, *in utero* fMRI

## Abstract

While the visuomotor system is known to develop rapidly after birth, studies have observed spontaneous activity in vertebrates in visually excitable cortical areas already before extrinsic stimuli are present. Resting state networks and fetal eye movements were observed independently *in utero*, but no functional brain activity coupled with visual stimuli could be detected using fetal fMRI. This study closes this gap and links *in utero* eye movement with corresponding functional networks. BOLD resting-state fMRI data were acquired from seven singleton fetuses between gestational weeks 30–36 with normal brain development. During the scan time, fetal eye movements were detected and tracked in the functional MRI data. We show that already *in utero* spontaneous fetal eye movements are linked to simultaneous networks in visual- and frontal cerebral areas. In our small but in terms of gestational age homogenous sample, evidence across the population suggests that the preparation of the human visuomotor system links visual and motor areas already prior to birth.

## INTRODUCTION

Postnatal sensory development is critical (Lewis and Maurer, 2005), as external stimuli shape the cortical architecture and its initial genetic roadmap (Bengoetxea et al., 2012). The nervous structures responsible for initial stimulus processing and evidence from animal studies suggest that already before the onset of vision, genetic factors, and spontaneous activity form the basis for subsequent topographic refinement of functional networks driven by experience. Vaidya and Gordon (2013) suggested that both genetic and experience-dependent maturational processes shape intrinsic connectivity networks.

Spontaneous neuronal activity is suspected to affect initial organization necessary for subsequent formation of more mature networks (Katz and Shatz, 1996). Recently, spontaneous retinal activity was described in mice *in vivo*, but the extent to which corresponding structured information is transmitted through the sensory system is still unknown (Ackman et al., 2012). Comparable activity has not yet been observed in humans. While substantial work exists on postnatal visual development in children, little is known about the events during the prenatal stimulus scarce period, when intrinsic activity is expected to dominate. Responses to visual stimuli were reported from as early as the 28th gestational week using neural electromagnetic activity measures acquired with magnetic resonance encephalography (MEG; Eswaran et al., 2004). *In utero* resting state networks (Schöpf et al., 2012; Thomason et al., 2013), and fetal eye movements (Birnholz, 1981; Woitek et al., 2013) were observed independently, but no visual stimulus response could be located in fetal functional magnetic resonance imaging (fMRI; Fulford et al., 2003). It is well established that resting-state fMRI is a powerful tool to study the organization of functional brain networks (Rosazza et al., 2012). The analysis of fMRI is not trivial and often requires data driven algorithms, such as independent component analysis [e.g., Schöpf et al. (2010a,b)]. The emergence of the visuomotor system is particularly informative regarding the precursory period of post-natal vision. It involves the relationship of intrinsic and extrinsic components suspected to shape the subsequent development of perception. The interaction of active eye movements and vision remains central in adult perception. In this work we investigated the relationship between eye movements and functional activity *in utero*, to identify the corresponding functional networks present before birth.

Even-though the questions where functional visual activity occurs *in utero*, and how primary visual areas do respond to extrinsic stimuli are not yet answered, voxel based morphometry studies in congenitally blind subjects hint at brain areas that change due to the processing of extrinsic stimuli. While congenitally blind subjects exhibit lower cortical volume in both frontal areas (BA 44, 45) and visual areas (BA 17, 18; Ptito et al., 2008), compared to a control population, functional connectivity between frontal areas (parts of BA 44, 45, and 47) and occipital areas is stronger in early blind (Liu et al., 2007). This suggests an ambiguous relationship between associated intrinsic and extrinsic processing systems (Golland et al., 2007). It might indicate that an initial abundance in connectivity between such areas as those implicated for language and visual processing is pruned after vision onset. While this then results in a clearer separation between the two systems, the network as a whole is relevant during initial organization of functional architecture.

Here we use data driven methodology to first show that there exists a link between spontaneous eye movements and functional networks in the occipital brain regions associated with vision. Furthermore, we detected stable functional networks involving both occipital and frontal regions.

## MATERIALS AND METHODS

### SUBJECTS

Seven singleton fetuses between gestational weeks (GW) 30–36 [mean: 32.86 (SD 1.81); mean age of mothers at MRI: 32.29 years (3.99 SD)] with no pathological brain development were scanned after informed consent was obtained from the mothers. The ethics committee of the Medical University of Vienna approved the study.

### IMAGE ACQUISITION

Magnetic resonance imaging was performed on a 1.5 T unit (Philips Medical Systems, Best, The Netherlands) using a SENSE (sensitivity encoding) cardiac coil with five elements. The pregnant women were examined in supine position, and neither contrast agents nor sedatives were administered. Measurements were performed using single-shot gradient-recalled echo-planar imaging (EPI). Fifteen axial slices with slice thickness of 3 mm were acquired with a matrix size of 144 × 144, FOV of 250 × 250 mm and TE/TR of 50/1000 ms and a flip angle of 90°. Axial slices were positioned perpendicular to the fetal brainstem. Two experienced neuroradiologists (GK and DP) evaluated the MR images.

### TRACKING OF EYE MOVEMENTS RELATIVE TO HEAD AXIS

**FIGURE 1:**
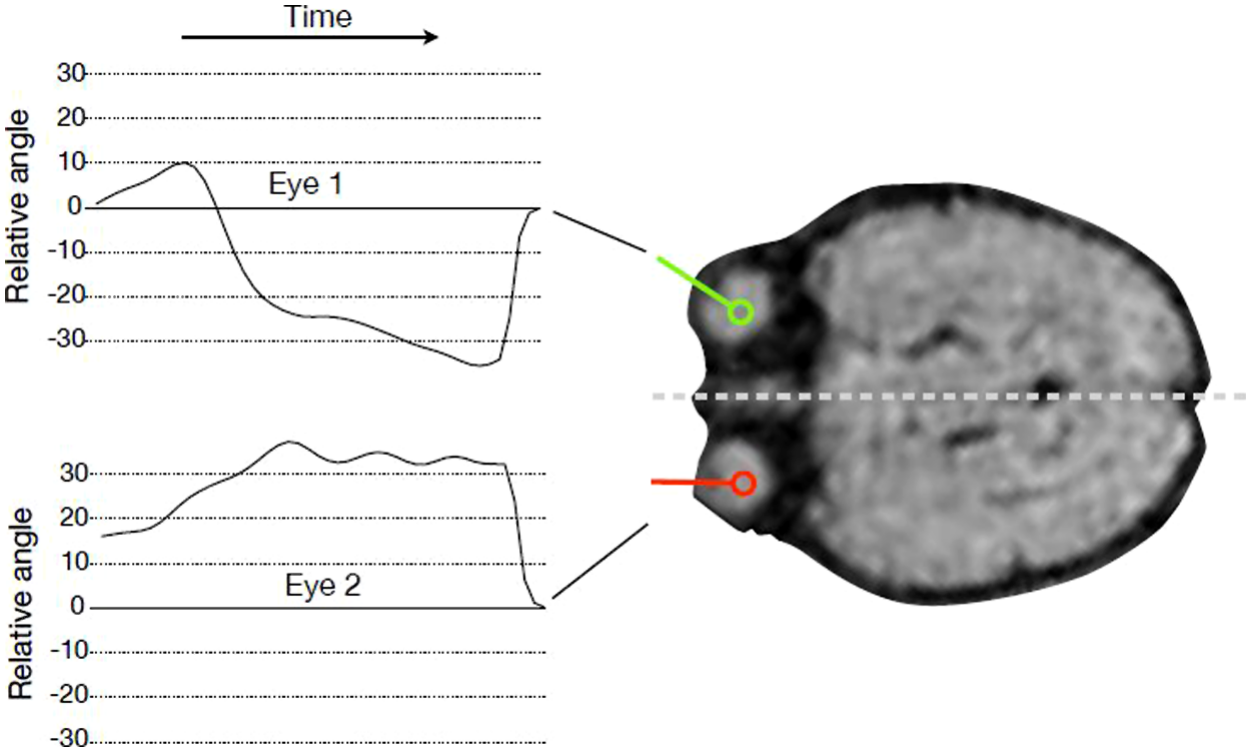
**Tracking eye movements over time.** For every time point in the fMRI sequence, the angle of gaze direction relative to head orientation was recorded for every subject and both eyes.

**FIGURE 2:**
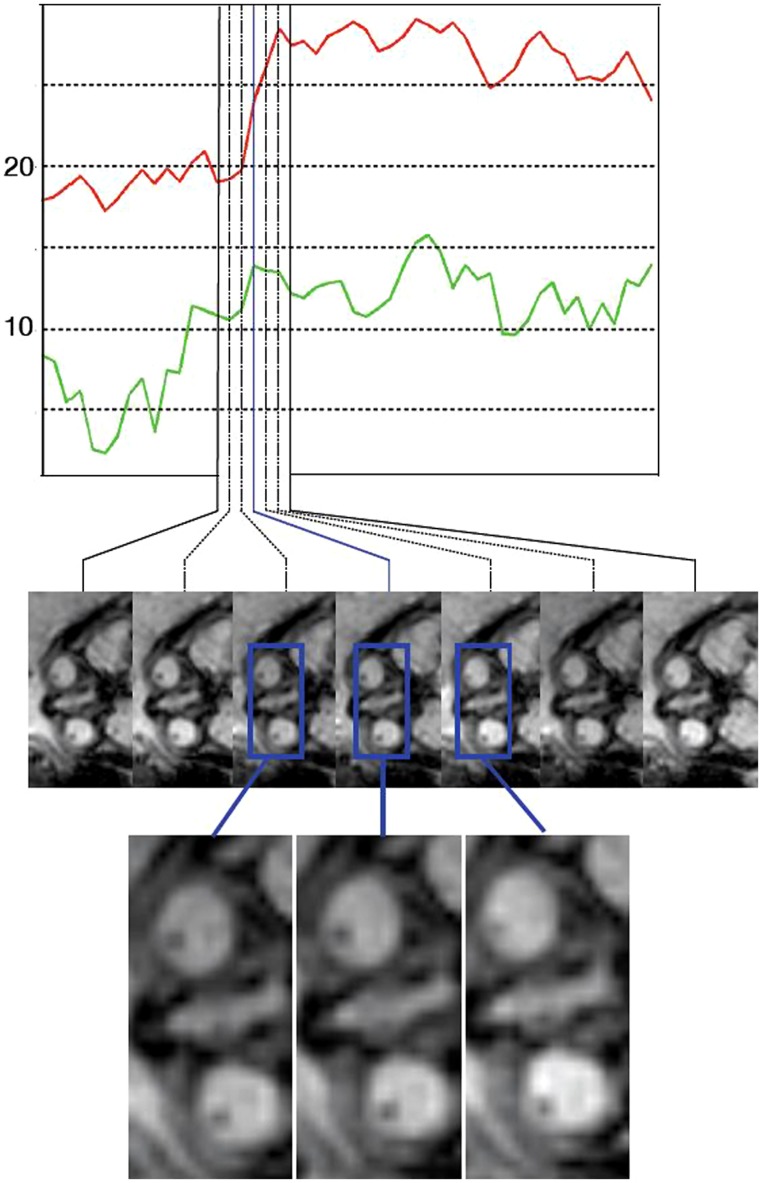
**An example of an eye movement sequence.** The top plot shows the measures eye angles relative to the head axis (red for eye 1 and green for eye 2). The images below show the imaging data depicting the eyes at the corresponding time points. The eye movements are in the range of 5–7° in this sequence, and therefore qualitative analysis by a reader is difficult.

We tracked the movements of both fetal eyes computationally based on the fMRI data (see **Figure 1**). First, we identified the MR slice that contained the eyes, and then pixels were classified by means of a random forest classifier (Breiman, 2001) to assign each pixel a probability of being part of an eye. Based on this probability map, eye center locations were estimated, and the segmentation of the eye was refined by mathematical morphology operations. The lens was detected along the eye border, and the view direction was calculated as the direction from eye center to the lens center. The head axis was defined as the symmetry axis between the two eyes. Relative eye angles were calculated as the difference between each eye angle (left and right) and the head central axis. Eye positions and relative eye angles were calculated for each frame in the MRI sequence resulting in a sequence of relative angles for the both eyes for each fMRI frame (see **Figure 2**).

Based on the eye tracking sequences we create regressors and event indicators defined as explanatory variables that reflect the onset of eye movement. Eye movement events are defined as the time points when the eye movement is initiated. We obtain these points by marking peaks of the derivative of the absolute value of the first derivative of the relative eye angles. These are time points when the change of relative eye angle accelerates. We discard peaks below a value of 1.4, and used the time points of the remaining peaks as event-indicators. For each eye this vector is then convolved with a standard hemodynamic response function corresponding to the TR of the fMRI sequence to obtain the final explanatory vector for each eye.

$$e:↔\left(d\left(|d(\alpha_{1})/dt|\right)/dt>t\right)\vee \left(d\left(|d\left(\alpha_{2}\right)/dt|\right)/dt>t\right)$$

Description of main movement regressor: eye movement onset left/right jointly.

### MOTION CORRECTION AND REGISTRATION

Image preprocessing was performed with FSL (FMRIB’s Software Library, www.fmrib.ox.ac.uk/fsl) including motion correction as implemented in MCFLIRT version 5.5 (Oakes et al., 2005).

### NUISANCE REGRESSORS

To lessen the confounding effects of non-neural signal fluctuations, we used a GLM based procedure to orthogonalize the fMRI signal to nuisance signals. Typically, such signals are originating from fetal head or maternal respiratory motion, and physiological noise, such as cardiac effects. We utilized the CompCor approach (Behzadi et al., 2007) which uses anatomical priors and principal component analysis to define non-neural signal. Fetal specific atlases were used to derive the CSF and WM compartments. We used CompCor regressors from an eroeded white matter mask, and from a region surrounding the cortical surface, including the CSF and the skull. For each region average signal and the most dominant six principal components were used as regressors. Furthermore derived nuisance regressors from the head and eye tracking information: (1,2) head position, (3,4) change of head position (x,y), (5) head angle, (6) change of head angle. The measurements from head tracking were convolved with a Gaussian filter, with SD 2.

### ANALYSIS

Single subject independent component analysis was performed using probabilistic ICA as implemented in Multivariate Exploratory Linear Decomposition Optimized into Independent Components (MELODIC) version 3.10, a part of FSL (FMRIB’s Software Library, www.fmrib.ox.ac.uk/fsl), using FastICA (Beckmann and Smith, 2004). The number of sources was estimated from the data by maximizing the Laplacian approximation to the Bayesian evidence of the model order (Minka, 2000; Beckmann and Smith, 2004). For the optimization of the non-Gaussian sources, contrast function, and convergence thresholds, as suggested by Hyvärinen et al. (2001), were used. Estimated component maps were divided by the SD of the residual noise and thresholded by fitting a mixture model to the intensity values histogram (Beckmann and Smith, 2004). Cross correlation coefficients of the derived independent components with all eye movement parameters were calculated.

## RESULTS

**FIGURE 3:**
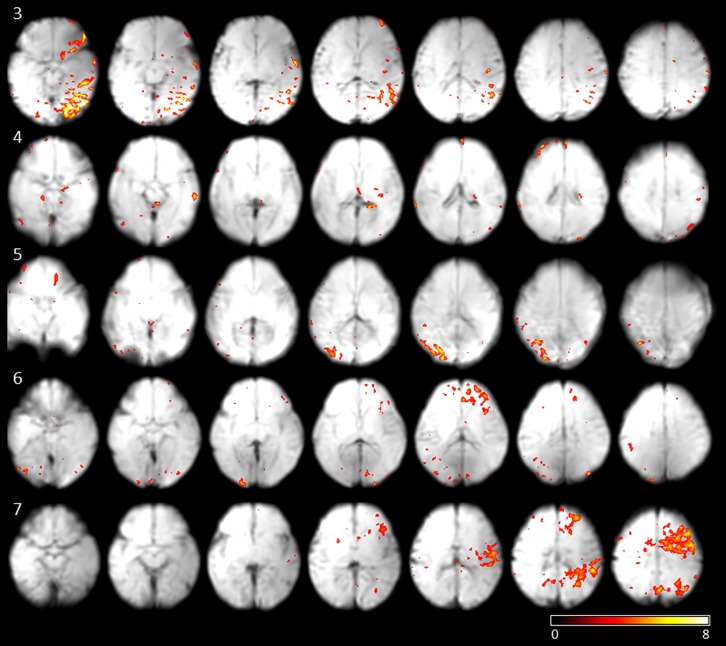
**Results of subjects 3–7 of the independent components with the maximum correlation coefficients with the eye movement on-set regressor derived from eye tracking.** Maps are overlaid on individual mean EPIs.

To investigate if the networks active at movement onsets are stable, and can be detected from the data regardless of the eye movement, we performed ICA on the fMRI data. On average 13 independent components were estimated per subject (SD 4.03). Correlation of single-subject component time courses with the eye movement regressor was calculated. The independent component with maximum correlation coefficients for each subjects were evaluated [mean correlation coefficient: 0.33 (SD 0.07)]. The networks showing the highest correlation with fetal eye movement (see **Figure 3**) include association related areas as the angular gyrus, the inferior parietal gyrus, the superior frontal gyrus, as well as a primary visual area the medial occipital gyrus and motion associated function.

## DISCUSSION

The present results indicate a relationship between fetal eye movements and activation in visual brain areas, motor areas, and orbitofrontal areas *in utero*. The observed functional networks correlating to visuomotor activity may offer a new perspective of the time period in which the orbitofrontal prepares for extrinsic sensory processing. The presence and relationship of the reported functional networks between visual and frontal areas in fetuses may prepare the developing brain for the processing of visual patterns as a precursor for the subsequent postnatal stimulus driven development of visual perception. Although observations are based on a limited number of subjects, the results were consistent across the population.

### FETAL EYE MOVEMENTS

Previous work on *in utero* fMRI provides insight in the developing brain ranging from stimulus processing (Schöpf et al., 2011) to intrinsic resting-state activity (Schöpf et al., 2012; Thomason et al., 2013). The observation of brain networks *in utero* suffers from the uncertainty of the status of the fetus at rest or asleep, which cannot be controlled. According to the categorization of fetal behavioral states from Romanini and Rizzo (1995), periods of “quiet sleep” or “quiet awake” can only be differentiated upon fetal eye movement. Four fetal eye movement patterns were initially characterized based on early ultrasound observations (Birnholz, 1981): Type I eye movements were described as single, transient deviations consisting of a bulb deviation, and a slower return back to the resting position, single but prolonged eye movements as Type II, complex sequences of eye movements to different directions without periodicity as Type III, and repetitive nystagmoid eye movements as Type IV. Additionally splitting of Type I eye movements into Type Ia (fast deviation, slower reposition) and Type Ib (fast deviation, equally fast reposition) was based on eye movement observation during MRI measurements (Woitek et al., 2013). It has already been shown that MRI sequences provide a valuable tool for visualizing and categorizing fetal eye movements (Brémond-Gignac et al., 1997; Woitek et al., 2013). While this method was initially developed to serve as an indicator for ocular development and as an indirect biomarker to detect malformations affecting the brainstem, our study investigates for the first time the networks responsible for the processing of eye movement *in utero*, enabling the purest form of a natural self induced stimulus, eye movement.

### FUNCTIONAL SENSORI MOTOR NETWORKS *IN UTERO*

We did not observe activations in the frontal eye field, which is known to be related to saccadic eye movements. As saccades are very fast (20–400 ms), we do not expect them to be correlated with the MRI tracked motion events due to the limited temporal resolution of the MRI eye tracking.

Slow pursuit or gazing movements (looking at directions intentionally or following a moving object) are controlled with involvement of deep-parieto-occipital cortex. Although we cannot confirm on intentional focusing *in utero,* we do see parietal–occipital activity in four out of seven cases. It is known from studies on macaques that the parieto-occipital cortex shows activation during self-generated eye movements (Law et al., 1998) and plays a crucial role in encoding extrapersonal visual space (Galletti et al., 1995). As we did not provide the fetus with visual stimuli, the activation we observed in the parietal–occipital area might hint at the generator of intrinsic eye movements in human fetuses.

There is only little motor activity and little involvement of the supplementary motor cortex or the typical frontal eye field (see below). These results are in line with findings in macaques reporting on fetal eye motion mainly governed by brainstem and midbrain processes. They suggest supratentorial control of eye motion gaining functional importance during brain development.

The networks we observed in fetuses differ from those known in the adult brain, and do not include a number of regions typically associated with eye movements in adults. Instead, they include areas identified with extrinsic stimulus processing (V1 in subjects 3,4,5,6) and motor control (subjects 3,4,6,7). So far no visual stimulus fMRI response activity *in utero* could be located (Fulford et al., 2003).

### THE FETAL- VERSUS THE ADULT BRAIN

Most experiments investigating sensory systems *in utero*, as for example visual and auditory systems, were constituted based on block length and stimulus sequences of adult paradigm settings, which might be a poor fit for fetal function and could explain the non-detection of activity patterns in, e.g., the primary sensory areas [for a review see Schöpf et al. (2011)]. The lack of previous localizations in primary sensory areas as a response to sensory stimuli strongly indicates that conventional fMRI paradigms used in adult settings do not capture the fetal brain settings. In light of (Dehorter et al., 2012) proposition that the developing brain is not a small adult brain but that the voltage- and transmitter-gated currents act like network driven patterns following a developmental sequence, the present results point to endogenous activity in the developing cortex as modulating the formation of functional units in the sensorimotor system. Instead of linking primary sensory activity to external stimuli, they evidence a link to spontaneous eye motion as part of the sensorimotor system. The function of these early patterns is to enable heterogeneous neurons to fire and wire together and prepare for subsequent stimulus driven development rather than to code specific modalities at this point.

Colonnese et al. (2010) already observed the change of functional units and a switch to mature visual response shortly before delivery in humans based on recordings from the visual cortex of preterm human infants. They proposed a “bursting” period of visual responsiveness during which weak retinal information is amplified by endogenous network oscillations, which in turn enable a primitive form of vision. Colonnese et al. (2010) concluded that an intrinsic program that is able to switch cortical responses in anticipation of patterned vision operates early visual developmental processing.

### LIMITATIONS

A constraining factor of the presented work is the limited sample size. According to this issue results are based on a single subject level analysis. As the sample was very homogenous with regard to gestational age this setting was chosen specifically to abstain from technical nuisance introduction emerging from registration to a template, which could influence the quality of single eye movement trackings. On the other hand, this homogeneity of developmental age does not allow for the analysis of eye movement related networks across gestational development, which will be of interest in future investigation. Furthermore, an important open question regarding our understanding of the hemodynamic developmental trajectory across *in utero* development, could be addressed by model driven data analysis.

## Conflict of Interest Statement

The authors declare that the research was conducted in the absence of any commercial or financial relationships that could be construed as a potential conflict of interest.
